# Digital CBT-I in Comorbid Insomnia and Depression: Clinical Outcomes From a Pragmatic Randomized Controlled Trial

**DOI:** 10.1155/da/2171041

**Published:** 2025-05-26

**Authors:** Jennifer Schuffelen, Leonie F. Maurer, Annika Gieselmann

**Affiliations:** ^1^Department of Clinical Psychology, Institute of Experimental Psychology, Heinrich Heine University, Universitätsstraße 1, Düsseldorf 40225, Germany; ^2^Mementor DE GmbH, Karl-Heine-Straße 15, Leipzig 04229, Germany

**Keywords:** cognitive behavioral therapy, depression, digital therapy, insomnia, randomized controlled trial

## Abstract

Depression affects 8.1% of the German population annually, yet many patients remain resistant to conventional treatments. Given that 85% of individuals with depression also experience chronic insomnia, sleep may represent both a contributing and modifiable treatment factor. This study examines whether adding a fully automated digital cognitive behavioral therapy for insomnia (dCBT-I) to care-as-usual (CAU) improves depressive symptoms. Participants with comorbid depression and insomnia were randomized to either the intervention group (dCBT-I) or the waiting group (WLC). The intervention was delivered via a mobile app or web platform, consisting of 10 sequential core modules based on evidence-based CBT-I techniques. Assessments took place at baseline, 12- and 24-weeks post randomization. The primary outcome was the severity of depressive symptoms (Patient Health Questionnaire-9; PHQ-9). Secondary outcomes included insomnia severity, daytime sleepiness, fatigue, well-being and mechanistic effect measures. Linear mixed models were calculated to determine between-group effects. A total of 140 participants (120 women, mean age: *M* = 39.76 ± 11.65 years) were randomized to dCBT-I (*n*=70) or WLC (*n*=70). Large treatment effects at 12- and 24 weeks showed significant reductions in depressive symptoms (−3.34 and −2.83; *p*s <0.001; *d*s = 0.66–0.78) in the dCBT-I group. Treatment effects in favor of dCBT-I were also found for insomnia severity (*d*s = 1.46–1.94) and most secondary outcomes (*d*s = 0.33–1.14). This study demonstrates that digital dCBT-I can be effective not only for individuals with primary insomnia but also for those with depression. These findings align with previous research, highlighting the crucial role of sleep disturbances in depression management. Moreover, the effects remained stable even in the heterogeneous sample investigated in this study, reinforcing the robustness of dCBT-I across diverse patient groups. Thus, dCBT-I emerges as a promising adjunctive treatment. Considering these findings, it is essential to explore the integration of sleep-focused interventions into standard depression treatment.

**Trial Registration:** German Clinical Trial Registry identifier: DRKS00030919

## 1. Introduction

Depression is one of the leading causes of work disability worldwide [1]. In Germany, its 12-month prevalence is 8.2% [2], contributing to approximately €8.7 billion in direct healthcare costs in 2015, representing 19.6% of all mental health-related medical expenses [3]. Despite effective treatments, depression has a high recurrence rate [4], with severity and relapse risk increasing after each subsequent episode [5, 6].

Primary care for depression in Germany is typically provided by general practitioners, involving antidepressant medication, psychotherapy, or a combination of both. [7]. However, effect sizes for these treatments are only small to moderate [8] and antidepressants are increasingly associated with side effects [9]. More than a third of patients do not respond to treatment, and outcomes have not improved in decades [10]. Although patients prefer psychotherapeutic approaches, access remains limited, with an average waiting time of 19.9 weeks [11]. Consequently, innovation is needed in terms of new treatment goals, forms of delivery and optimization.

Clinical and experimental studies show that the disruption of sleep plays a key function in the development and expression of psychological symptoms. The most common sleep disorder, insomnia disorder, is defined as chronic difficulties in initiating and maintaining sleep, accompanied by daytime impairment and diagnosed according to standard classification systems. Around 85% of patients with depression fulfill the criteria for insomnia disorder [12], while 93% report experiencing at least some insomnia symptoms, such as difficulties falling or staying asleep [13]. A bidirectional relationship between depression and insomnia disorder is well established [14] and recent research further supports the notion that insomnia disorder serves as a transdiagnostic treatment target [15]. Moreover, prospective studies indicate that insomnia disorder often precedes the onset of depression [16] and exacerbates depressive symptoms, such as negative affect, anhedonia, lethargy, concentration problems, and suicidality [17, 18].

Evidence from clinical studies shows both that the severity of insomnia symptoms at the beginning of depression treatment is associated with attenuated treatment success and that insomnia is one of the most common symptoms that persists after successful depression treatment and is also associated with relapse into depression [19, 20]. Treating insomnia symptoms in patients with depression may, therefore, enhance therapeutic effects and reduce recurrence risk. [21].

To treat symptoms of insomnia, cognitive behavioral therapy (CBT-I) is recommended as first-line treatment [22], which shows large treatment effects for insomnia severity (Hedges' *g* = 0.98) [23], and medium-to-large effect sizes for depression [24, 25]. These results are also supported by a recent review stating that CBT-I is particularly recommended as a treatment for depression when antidepressants are not suitable due to side effects [26].

Despite its efficacy, traditional face-to-face CBT-I is not feasible for broad implementation due to resource constraints [27]. Digital cognitive behavioral therapy for insomnia (dCBT-I) provides a scalable, cost-effective alternative [28] and has been integrated into regular care for insomnia disorder in Germany since October 2020. While initial studies support dCBT-I's efficacy in populations with both insomnia disorder and depression [29–31], most trials lack diagnostic confirmation of depression and only measure depressive symptoms as secondary outcomes. Furthermore, given the unique integration of dCBT-I into regular care in Germany, it is important to acknowledge that the strong control mechanisms in randomized controlled trials inherently limit the generalizability of findings.

Altogether, our study aims to test whether adding an effective dCBT-I (somnio, mementor DE GmbH) to care-as-usual (CAU) for depression can improve depression symptoms in patients with depression and insomnia disorder, using less strict inclusion and exclusion criteria to enhance the generalizability of the findings. For this purpose, following primary and secondary hypotheses are set up.

1. Digital CBT-I reduces depression symptoms compared to the waiting group (WLC).
2. Digital CBT-I reduces self-reported insomnia severity compared to the WLC.
3. Digital CBT-I reduces daytime sleepiness compared to the WLC.
4. Digital CBT-I reduces fatigue compared to the WLC.
5. Digital CBT-I increases well-being compared to the WLC.
6. Compared to the WLC, digital CBT-I reduces the occurrence of negative emotions and increases the occurrence of positive emotions.
7. Digital CBT-I improves emotion regulation compared to the WLC.
8. Digital CBT-I improves self-efficacy compared to the WLC.

All hypotheses are tested both at post-intervention assessment (12-weeks, primary outcome) and follow-up assessment (24-weeks).

## 2. Methods

### 2.1. Study Design

We conducted a two-armed, randomized controlled trial, randomly assigning adult participants to either the intervention group or the WLC. The intervention group received dCBT-I, somnio, mementor DE GmbH for 12 weeks, while the WLC group received no intervention during this period but gained access to dCBT-I after the 24-week follow-up. Both groups continued to have access to CAU. Main assessments took place before the start of the intervention period (baseline), 12 weeks post randomization (post-intervention) and 24-weeks post randomization (follow-up). The study was conducted in Germany, approved by the Ethics Committee of Heinrich Heine University Duesseldorf, and preregistered at the German Clinical Trials Register (Deutsches Register Klinischer Studien; DRKS).

### 2.2. Participants and Procedure

Recruitment took place between January and May 2023 through advertisements in social media networks (primarily Facebook and Instagram) and mailouts. Participants had to meet the following inclusion criteria: (1) minimum age of 18, (2) current depressive disorder, operationalized by a diagnostic interview and Beck Depression Inventory (BDI) >8 [32], (3) current insomnia disorder, operationalized by a diagnostic interview and Insomnia Severity Index (ISI) ≥10 [33], (4) confident use of digital devices (smartphone, tablet, and computer), and (5) stable internet access. Participants could not be included in the study if they met any of the following exclusion criteria: (1) presence of bipolar disorder or psychosis, (2) presence of another sleep disorder, (3) regular consumption of alcohol (≥ 3 glasses daily for at least 3 weeks), use of cannabis (≥ 1 a week) or other illegal drugs, (4) pregnancy, (5) suicidality, (6) less than four entries in the baseline sleep diary.

Interested participants were directed to an online screening via SoSci Survey (SoSci Survey GmbH). After reading the patient information and providing consent, they completed a preliminary eligibility check, whereupon the inclusion and exclusion criteria for the study were checked. Potentially suitable participants were then asked to provide their contact details so that they could be contacted for a telephone interview. The telephone interview was used to explain the study in a personal conversation, clarify open questions, discuss the further study procedures, and conduct a clinical interview, which again checked the inclusion and exclusion criteria to ensure the suitability of the participants. In this interview, the diagnoses of insomnia and depression were confirmed. The interviews were conducted by master's students nearing the completion of their studies, under the supervision of a licensed psychotherapist.

Eligible participants then received a link to the baseline assessment, where they formally consented to the study and completed questionnaires. They were also required to complete an online sleep diary daily for 1 week. Only participants with at least four diary entries were randomized into either the intervention or WLC group. The intervention group received immediate 12-week access to dCBT-I via *somnio* (mementor DE GmbH). The WLC group entered a waiting period. Both groups were encouraged not to interrupt the utilization of their regular medical care. Further assessments took place at 12 weeks (post-intervention) and 24 weeks (follow-up), each including a 1-week online sleep diary. After the follow-up, study participation concluded, and WLC participants gained access to dCBT-I. The participants were not financially compensated, but all received *somnio*, which is a certified medical product in Germany (mementor DE GmbH).

### 2.3. The dCBT-I Intervention

Participants in the intervention group were instructed to begin the dCBT-I *somnio* (mementor DE GmbH) immediately after randomization. *somnio* comprises ten core modules, which are based on the elements of face-to-face CBT-I manuals, incorporating key components, such as psychoeducation, relaxation techniques, stimulus control, bedtime restriction, and cognitive therapy. The modules are unlocked sequentially by entering diary entries and completing previous modules. To support adherence and engagement, *somnio* includes a fully automated notification and reminder system that sends regular prompts via push notifications or email. These reminders encourage users to complete their daily sleep diary, continue previously started modules, or begin new ones, based on individual progress. Additional follow-up modules consolidate the knowledge acquired and reduce the risk of relapse. The content is delivered fully automated by an interactive avatar that guides the participants through the dCBT-I (See [34] for a more detailed overview). The effectiveness of *somnio* has so far been demonstrated in three randomized controlled trials [34–36]and a retrospective user data analysis of regular care patients [37]. Furthermore, a subgroup analysis of participants with high depressive baseline scores provided preliminary indications of the effectiveness of *somnio* in cases of comorbid depression [31].

### 2.4. Randomization and Masking

After completing the baseline assessment, participants were randomly assigned (1:1) to dCBT-I + CAU or WLC + CAU using a validated randomization program (*sealedenvelope.com*, London, UK). Stratification was performed according to age (18–39 and ≥40), gender (male and female), and intake of sleep medication (yes and no), with varying block sizes to ensure an equally balanced group distribution. A member of the study team, who had no contact with the participants during the entire duration of the study, managed the randomization sequence. Participants received information about the different groups and were informed about their group affiliation but were not provided with any information about the study hypotheses. While the study team was aware of allocations, they had no influence on the intervention or digital data collection. To minimize bias, data was blinded before analysis.

## 3. Measurements

### 3.1. Primary Outcome

The severity of depressive symptoms was measured using the Patient Health Questionnaire-9 (PHQ-9) [38]. It consists of nine items on a 4-point Likert scale from 0 (*not at all*) to 3 (*nearly every day*) to measure depressive symptoms in the last 2 weeks. Items are summed up to a total score ranging from 0 to 27, whereby higher values indicate higher depressive symptom severity. The PHQ-9 has shown good psychometric properties in medical settings [39–41] and in general population [42].

### 3.2. Secondary Outcomes

#### 3.2.1. Insomnia Severity

Self-reported insomnia severity was measured using the ISI [33]. With seven items on a 5-point Likert scale ranging from 0 (*not at all*) to 4 (*extremely*), the ISI asks about the sleep behavior of the previous 2 weeks. The items are added together, yielding a total score between 0 and 28, with higher scores indicating a greater insomnia severity. The ISI has shown very good internal consistency in the past [43–45].

#### 3.2.2. Daytime Sleepiness

Daytime sleepiness was measured using the Epworth Sleepiness Scale (ESS) [46, 47]. Eight items on a 4-point Likert scale, ranging from 0 (*no chance of dozing*) to 3 (*high chance of dozing*), were used to assess the probability of falling asleep during everyday situations. Items are summed up to a total score ranging from 0 to 24, with higher scores indicating greater daytime sleepiness. The ESS has proven to be a wide-validated measuring instrument with good internal validity (*α* ≥ 0.73–0.86) [48].

#### 3.2.3. Fatigue

Fatigue was measured using the Fatigue Severity Scale (FSS) [49]. It consists of nine items that measure the severity of fatigue over the past week on a 7-point Likert scale from 1 (*strong disagreement*) to 7 (*strong agreement*). A higher mean value indicates greater perceived fatigue. The FSS is a valid instrument for measuring fatigue [50].

#### 3.2.4. Well-Being

The World Health Organization-Five Well-being Index (WHO-5) [51, 52] was used to assess mental well-being. The questionnaire comprises 5 items on a 6-point Likert scale, ranging from 0 (*none of the time*) to 5 (*all the time*), with higher values indicating increased well-being. A score below 13 may indicate a diagnosis of major depression. The WHO-5 demonstrates excellent psychometric properties [53].

#### 3.2.5. Emotional State

The Positive and Negative Affect Schedule (PANAS) [54, 55] was used to record the emotional state of the last weeks. The PANAS uses 20 adjectives on a 5-point Likert scale from 1 (*not at all*) to 5 (*extremely*) to record positive and negative affective states. A mean value is calculated for each dimension, with higher values indicating higher emotionality. The PANAS has so far proven to be a valid instrument in both nonclinical [56] and clinical samples [57–59].

#### 3.2.6. Emotion Regulation

The short version of the Response Style Questionnaire (RSQ) [60] was used to measure emotion regulation. The RSQ measures coping styles, which are associated with the presence of depressive mood. It consists of a total of 23 items, which are measured on a 4-point Likert scale from 1 (*almost never*) to 4 (*almost always*). A distinction is made between the three subscales *self-focused rumination*, *symptom-focused rumination*, and *distraction*. A total score is derived for each subscale by summing up the related items. The RSQ achieves satisfactory psychometric properties and thus proves to be a reliable measuring instrument [60].

#### 3.2.7. Self-Efficacy

The Perceived Competence Scale (PCS) [61] was adapted for the specific scope of this study to measure the perceived self-efficacy in relation to the experienced sleep disturbances. The questionnaire consists of four items on a 7-point Likert scale. The values range from 1 (*strongly disagree*) to 7 (*strongly agree*), with an average of all items being used to calculate a total mean score. A higher mean score indicates a higher self-rated self-efficacy. In the past, the PCS has proven to be a reliable measurement tool [61].

#### 3.2.8. Sleep Diary

A 1-week sleep diary was used at baseline assessment, posttreatment assessment and follow-up assessment. It asks about the sleep parameters of the previous night, that is sleep onset latency (SOL), wake after sleep onset (WASO), total sleep time (TST), time in bed (TIB) and sleep efficiency (SE), as well as sleep medication intake. The sleep diary was included in the analysis if at least four entries were entered by the participants in the respective week.

#### 3.2.9. *Adherence*, *Treatment Satisfaction, and Adverse Events*

Adherence to the intervention was operationalized using the user data within the intervention, whereby the number of completed modules was examined. In addition, three 5-point Likert scales, ranging from 1 (*not at all*) to 5 (*completely*), were used to measure the participants' satisfaction with the intervention, the fulfillment of expectations of the intervention and the conscientiousness with which the modules were completed. Participants of both groups were instructed at the beginning of the study to contact a member of the study team in the case of adverse events.

### 3.3. Data Analysis

The sample size was calculated using G^*∗*^Power [62]. The primary outcome was the severity of depressive symptoms at 12 weeks post randomization. Assuming that this originates from a normally distributed population, the hypothesis needed to be tested in a parallel group design with a *t*-test for independent samples. In order to achieve a statistically significant group difference of SMD = 0.65 [31] with a power of 1-*β* = 0.9 and a two-sided probability of error of *α* = 0.05, *n*=102 participants were required. Further assuming a 20% drop out of the study, the power analysis yielded a total sample size of *n*=122 participants. All analyses were conducted using SPSS.29 (IBM). In accordance with CONSORT guidelines, available data from all randomized participants were analyzed following the intention-to-treat principle [63, 64].

Linear mixed-effects regression models with fixed effects of group and time were adjusted for between-group comparisons of primary and secondary outcomes, with missing data taken into account. Outcomes at 12- and 24-weeks were included as a response. To adjust for baseline measurements, the baseline value was entered as a covariate and a participant-specific random intercept was added to account for repeated measures [65]. A time point by group interaction was included to estimate treatment effects at each time point. The covariance structure was set to unstructured.

Cohen's *d* was used to determine between-group effect sized and was calculated by dividing the adjusted mean difference by the standard deviation of both groups at baseline [66]. For continuous sleep diary data, responses were entered as averages of 1-week periods and compared to late treatment (weeks 12 and 24) adjusting for values before the beginning of the treatment (week 0). Responder (≥50% reduction in PHQ-9 score) and remission rates (PHQ-9 score < 5) were calculated for the primary outcome to determine clinical significance [67]. With regard to the secondary outcome of self-reported insomnia severity, responder (ISI reduction ≥ 8) and remission rates (ISI total score < 8) were calculated [33, 45]. Between-group differences of these dichotomous outcomes were analyzed using Pearson chi-squared tests and effect sizes were quantified using phi. Descriptive statistics are presented by unadjusted means (*M*) and standard deviations (*SD*) for continuous outcomes, and frequencies for binary outcomes.

## 4. Results

**Figure 1:**
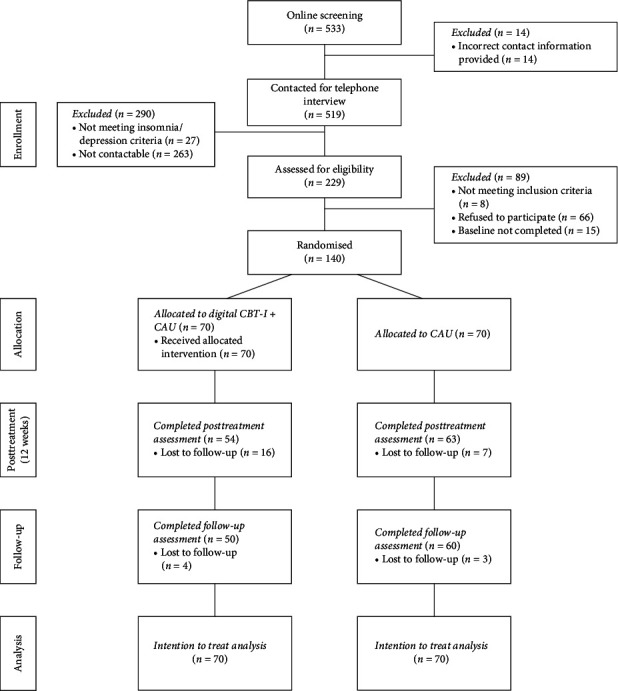
Participant flow.

Overall, *n*=533 potential participants completed the online screening, with *n*=14 provided incorrect contact information, resulting in *n*=519 being contacted for a telephone interview. Out of these, *n*=290 participants were excluded, primarily due to nonresponsiveness, leaving a total of *n*=229 participants who were considered eligible for the baseline assessment. Once the predefined sample size of 122 was reached, recruitment was halted. However, for ethical reasons, all participants assessed as eligible by that point were included, resulting in a final sample of *n*=140 randomized participants. The dropout rate at the post-intervention assessment was 16.43%, increasing to 21.43% by the follow-up assessment, reflecting slight additional attrition over time. The participants flow is shown in Figure 1.

On average, the participants were 39.76 ± 11.65 years old, with the majority being female (85.7%). Most had completed either a university degree (40.7%) or an apprenticeship (32.9%).

Regarding inclusion criteria, participants had an average PHQ-9 score of 14.63 ± 5.40, indicating mild to moderate depression. Furthermore, with an average ISI score of 16.84 ± 5.15, the participants exhibited moderate insomnia. Detailed demographic and baseline characteristics of both groups are provided in Table 1.

### 4.1. Primary Outcome

**Figure 2:**
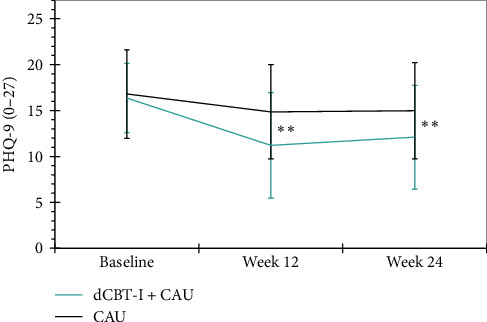
Changes in primary outcome (depression), across both groups and all assessments. Unadjusted means (± 1 SD) are presented for both groups. Statistical group differences are derived from linear mixed models and represented by a double asterisk (*⁣*^*∗*^*⁣*^*∗*^*p* < 0.001).

*Depressive symptoms*. The primary aim of this study was to investigate whether the implementation of dCBT-I, in addition to CAU, and in comparison to a WLC group, can lead to a reduction in depressive symptoms in a sample of participants with diagnosed depression. The results of the linear-mixed-model revealed medium between-group effects in favor of the intervention group at both 12- (*p* < 0.001, *d*=−0.78) and 24-weeks (*p* < 0.001, *d*=−0.66). On average, depressive symptoms were reduced by −3.34 and −2.83 points respectively compared to the WLC group. See Figure 2 for a graphical representation. After 12-weeks, 30% (*n*=21) of the participants in the intervention group could be classified as responders, compared to 8.6% (*n*=6) of the WLC group, *χ*^2^ (1, *n*=117) = 14.13, *p* < 0.001, *φ* = -0.35. After 24 weeks post randomization, 21.4% (*n*=15) of the intervention group and 4.3% (*n*=3) of the WLC group were still considered responders, *χ*^2^ (1, *n*=110) = 12.46, *p* < 0.001, *φ* = -0.34. With regard to remission rates, 5.7% (*n*=4) in the intervention group and 1.4% (*n*=1) in the WLC group achieved the criterion after 12-weeks post randomization, *χ*^2^ (1, *n*=117) = 2.41, *p*=0.121, *φ* = -0.14. After 24 weeks post randomization, 5.7% (*n*=4) of the intervention group and 2.9% (*n*=2) of the WLC group still met the criteria for remission, *χ*^2^ (1, *n*=110) = 1.15, *p*=0.408, *φ* = −0.10. The results of the statistical analysis of the primary and secondary clinical outcomes are shown in Table 2.

### 4.2. Secondary Outcomes

**Figure 3:**
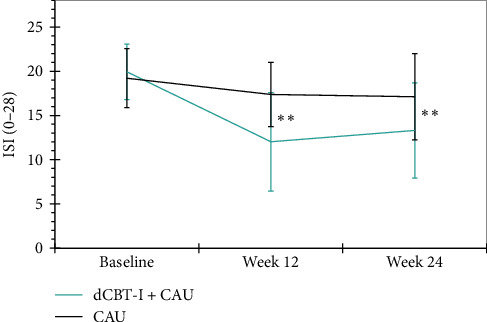
Changes in secondary outcome (insomnia severity), across both groups and all assessments. Unadjusted means (± 1 SD) are presented for both groups. Statistical group differences are derived from linear mixed models and represented by a double asterisk (*⁣*^*∗*^*⁣*^*∗*^*p* < 0.001).

*Insomnia symptoms*. There were large between-group effects in favor of the digital CBT-I group at 12- (*p* < 0.001, *d*=−1.94) and 24-weeks post randomization (*p* < 0.001, *d*=−1.46). This indicates that the severity of self-reported insomnia symptoms decreased sustainably as a result of the digital CBT-I intervention (Figure 3). Overall, 50% (*n*=35) of participants in the intervention group could be categorized as responders after 12-weeks in the between-group comparison, compared with 5.71% (*n*=4) in the WLC group, *χ*^2^ (1, *n*=115) = 45.27, *p* < 0.001, *φ* = −0.63. After 24-weeks post randomization, 34.29% (*n*=24) of the intervention group and 14.29% (*n*=10) of the WLC group were still classified as responders, *χ*^2^ (1, *n*=110) = 12.54, *p* < 0.001, *φ* = −0.34. Remission was achieved by 18.57% (*n*=13) of participants in the intervention group compared to 0% (*n*=0) in the WLC group at 12-weeks post randomization, *χ*^2^ (1, *n*=115) = 17.15, *p* < 0.001, *φ* = −0.39. After 24-weeks post randomization, 11.4% (*n*=8) of the intervention group and 5.7% (*n*=4) of the WLC group still met the criteria for remission, *χ*^2^ (1, *n*=110) = 2.44, *p*=0.136, *φ* = −0.15.

*Daytime sleepiness*. The analysis of daytime sleepiness revealed no significant effects between both groups at either timepoint (*p*=0.607 and *p*=0.721).

*Fatigue*. Between-group comparisons showed large treatment effects in favor of the digital CBT-I group after 12- (*p* < 0.001, d = −0.98) and 24-weeks (*p* < 0.001, *d*=−0.81) post randomization, suggesting a reduction in fatigue due to the digital CBT-I Intervention.

*Well-being*. The results of the linear mixed model showed large effect sizes at 12- (*p* < 0.001, *d* = 1.09) and 24-weeks (*p* < 0.001, *d* = 1.14) post randomization. The dCBT-I group showed improved well-being compared to the WLC group.

*Emotional state*. For the positive affect subscale, the linear mixed model showed medium to large effect sizes in favor of the dCBT-I group at 12- (*p* < 0.001, *d* = 0.56) and 24-weeks (*p* < 0.001, *d* = 0.98) post randomization and thus an increase in positive affect. A similar pattern of results was also found for the negative affect subscale. Both, after 12- (*p* < 0.001, *d*=−0.52) and 24-weeks (*p* < 0.001, *d*=−0.74) post randomization, the dCBT-I group showed reduced negative affects compared to the WLC group with medium effect sizes.

*Emotion regulation*. The effects on emotion regulation were calculated separately by subscale. The analysis of the *self-focused rumination* subscale did not reveal a between-group effect 12-weeks post randomization (*p*=0.594). After 24 weeks, however, there was a small effect in favor of the digital CBT-I group (*p*=0.013, *d*=−0.33). Between-group comparisons of the *symptom-focused rumination* subscale revealed small treatment effects in favor of the digital CBT-I group at 12- (*p*=0.005, *d*=−0.40) and 24-weeks (*p*=0.002, *d*=−0.46) post randomization. In contrast, no between-group effects were found for the *Distraction* subscale (*p*=0.925 and *p*=0.627).

*Self-efficacy*. No treatment effects were found regarding self-efficacy between the dCBT-I group and the WLC group after either 12- (*p*=0.932) or 24-weeks (*p*=0.764) post randomization.

*Sleep diary outcomes*. With regard to *sleep-onset latency* (SOL), no significant between-group effect was found either at 12-weeks (*p*=0.207) or at 24-weeks (*p*=0.880) post randomization. The linear mixed model revealed a reduction in WASO with small effects at 12-weeks (*p*=0.003, *d*=−0.29) and 24-weeks (*p* < 0.001, *d*=−0.44) post randomization. Small to moderate effects in favor of the treatment group were found in relation to the improvement in SE 12-weeks (*p* < 0.001, *d* = 0.63) and 24-weeks (*p*=0.004, *d* = 0.40) post randomization. The TST was not significantly reduced in the intervention group compared to the WLC after 12 weeks (*p*=0.111) post randomization. After 24 weeks post randomization, however, there was a significant increase in TST in the intervention group compared to the WLC group with small effect size (*p*=0.005, *d* = 0.36). In contrast, TIB was reduced in the intervention group compared to the WLC group 12-weeks post randomization with small effect size (*p*=0.002, *d*=−0.41). This effect was no longer significant 24-weeks post randomization (*p*=0.746). An overview of the between-group differences in sleep diary parameters is provided in Table 3.

Sleep medication entries showed that *n*=33 participants (dCBT-I = 18; WLC = 15) reported taking prescribed medication with sedating effects at baseline, *n*=29 (dCBT-I = 12; WLC = 17) at 12-weeks, and *n*=23 (dCBT-I = 10; WLC = 13) at 24-weeks post randomization. Over the counter remedies were taken by *n*=8 participants (dCBT-I = 5; WLC = 3) at baseline, *n*=7 participants (dCBT-I = 4; WLC = 3) at 12-weeks, and *n*=6 participants (dCBT-I = 3; WLC = 3) at 24-weeks post-randomization. Overall, there was a high rate of missing diaries at the post-intervention assessment (23.57%) and follow-up (30%). The rate of missing diaries was noticeably higher in the intervention group than in the WLC group at both time points (31.88% versus 15.71% and 37.68% versus 22.86%) and therefore need to be interpreted with caution.

*Adherence*, *treatment satisfaction and adverse events*. In total, 67.14% of participants in the dCBT-I group (*n*=47) completed at least half of the 10 core modules, with 37.14% (*n*=26) completing the last module. Overall, 34 (48.57%) participants who received dCBT-I stated that they were *satisfied* or *very satisfied* with the intervention. A further 11 (15.71%) indicated a *neutral* attitude towards satisfaction with the intervention. The majority stated that their expectations of the dCBT-I were *largely* or *completely* fulfilled (*n*=27, 38.57%). Sixteen participants (22.86%) stated that their expectations had been *partially* fulfilled and only 3 participants (4.29%) had not fulfilled their expectations of the intervention at all. Over half of the participants (*n*=37, 52.86%) stated that they had *largely* or *completely* conscientiously undertaken the intervention. In contrast, no participant (0%) stated that they had not completed the modules conscientiously at all. No adverse events were reported by the *n*=70 participants in the intervention group.

## 5. Discussion

The primary aim of this study was to examine whether adding dCBT-I to CAU for depression could reduce depressive symptoms compared to a WLC group. The majority of participants included in this study received or have received psychotherapeutic treatment as well as medication (Table 1) indicating that the present study sample has already received antidepressant treatment before the start of the study but still presented symptoms for depression and the need for further treatment.

It was shown that the addition of dCBT-I to CAU was superior to the WLC group (+ CAU) in reducing depressive symptoms with medium effect sizes at all timepoints (*d*s = −0.78 and −0.66). Indeed, effect sizes were comparable with those for psychotherapy for depression (Hedges *g* = 0.71, after adjustment for bias *g* = 0.53 [68] and in line with previous studies that have demonstrated positive effects of CBT-I on depressive symptoms [29, 30].

The strongest effects were observed for insomnia severity (*d*s = −1.94 and −1.46), which aligns with meta-analyses of digital CBT-I [69–71]. However, while the treatment was highly effective in reducing insomnia severity, the responder rate for insomnia (50%) and remission rate (18.57%) at 12 weeks were slightly lower than in a prior study using the same intervention (63.6% and 40.7%, respectively, [34]. When compared to a study sample with similar characteristics regarding depressiveness, comparable rates of remission of insomnia symptoms were achieved [72]. This suggests that the comorbid presence of two psychological disorders likely contributes to the observed differences in responder and remission rates, as dual pathology can make symptom remission more challenging to attain. Nevertheless, our results emphasize the importance of addressing insomnia symptoms in depression and support the hypothesis that insomnia plays a crucial role in the maintenance of depressive symptoms [17, 18].

Beyond insomnia and depression severity, dCBT-I significantly improved secondary outcomes, such as well-being and emotion regulation. Interestingly, the present study observed a notable difference between the outcomes for daytime sleepiness and fatigue, with no significant effect on daytime sleepiness but large treatment effects regarding fatigue (*d*s = −0.98 and −0.81). This discrepancy highlights the complexity of sleep-related daytime symptoms and suggests distinct mechanisms underlying these constructs. It could be argued that the fatigue measurement better captured the exhaustion related to depression than the measure of daytime sleepiness. This aligns with the idea that fatigue is heavily impacted by psychological factors, such as cognitions [73], which were likely improved through the intervention. In contrast, daytime sleepiness measures the physiological tendency to feel sleepy during the day [47]. Indeed, participants in this study seemed to have reported normal levels of daytime sleepiness at baseline (ESS score below nine) leaving limited room for measurable improvement.

The sleep diary showed mixed results overall, which did not always align with the strong improvements observed in other assessments, such as the ISI. Specifically, while we found large improvements in the ISI scores, the sleep diary indicated improvements only for WASO and SE, but not for SOL. However, these findings should be interpreted cautiously due to low diary completion rates, particularly at post-intervention (76.26%) and follow-up (69.78%). Only a small proportion of participants (25.18% in total) completed 7 days of sleep diary at all three assessments. It could be argued that this duration is not sufficient to provide reliable and valid results, because it is recommended that the diary should be kept for at least one to 2 weeks [74].

A key strength of this study is its focus on a clinically relevant, heterogeneous sample. Unlike many RCTs that apply strict inclusion criteria and yield highly selective samples, we intentionally used broader inclusion and exclusion criteria to enhance the generalisability of our findings. This approach reflects the real-world population more accurately, capturing individuals who often present with comorbid conditions and have already undergone conventional depression treatments but remain symptomatic. The integration of dCBT-I into routine healthcare in Germany further underscores the significance of these findings. In contrast to other countries where digital interventions are still largely experimental, Germany has fully incorporated dCBT-I into primary care through the Digital Health Applications (DiGA) framework. While our study expands on prior research by using a more diverse sample, further research is needed to assess dCBT-I's effectiveness in routine clinical settings. Furthermore, the choice of depression severity as the primary outcome measure in a sample with both depression and comorbid insomnia, emphasizes the bidirectional relationship between insomnia and depression, highlighting the potential of dCBT-I as an adjunct treatment for patients with residual depressive symptoms. This is crucial for clinical practice, where comorbidity is common, and addressing interconnected symptoms can lead to a more effective, holistic treatment approach, especially for those who have not fully benefited from typical therapeutic approaches.

However, limitations should also be considered. First, this study was open-label, meaning that participants in the WLC group had unrestricted access to CAU but did not receive an active intervention. As a result, they were aware of their allocation to the control group, which may have influenced subjective outcome measures, potentially introducing response biases [75]. Second, the high dropout rates in this study (16.43% and 21.43%) should also be mentioned. While those rates are in range of those to be expected in digital interventions that are offered without any human support [76], it raises questions about acceptance and adherence in clinical populations. Third, the recruitment strategy took place entirely online and may have targeted an internet-affine group and may not represent the wider population. Yet, this method was chosen to reach people throughout Germany who suffer from insomnia and depression.

Altogether, the present study demonstrated the beneficial effects of applying dCBT-I as an additional factor to regular care in a population with depression. The addition of an effective dCBT-I not only sustainably reduced the severity of insomnia and associated daytime symptoms compared to a WLC group, but also improved the severity of depression and other secondary depression-associated symptoms. Even though only a small proportion (5.7%) achieved remission, it is nevertheless a promising result that this supposedly treatment-resistant population could achieve symptom remission to a clinically significant extent. Furthermore, 30% (12 weeks) and 21.4% (24 weeks) responders were achieved, meaning that around one in three people benefit from the addition of dCBT-I to a clinically significant extent. Considering that insomnia is often not specifically treated in the presence of depression, there is high potential to improve outcomes in general and speed up the therapeutic process. Moreover, digital solutions should also be taken into consideration, particularly due to their high cost- and time-benefit efficiency and the potential to provide the treatment required more easily to a wider population.

## Figures and Tables

**Table 1:** Participant demographic and baseline characteristics.

Demographic variables			dCBT-I	WLC group
			*n*=70	*n*=70
Baseline characteristics				
Age, years, *M* (*SD*)			39.56 (11.31)	39.96 (12.07)
Female, *n* (%)			59 (84.3)	61 (87.1)
Symptom duration, years, *M* (*SD*)			6.17 (6.98)	8.66 (8.67)
Distinct trigger that caused symptoms, *n* (%)			21 (30.0)	19 (27.1)
Shared bedroom, *n* (%)			14 (20.0)	20 (28.6)
Shift work, *n* (%)			8 (11.4)	6 (8.6)
Children disrupting sleep, *n* (%)			5 (7.1)	2 (2.9)
*Psychotherapy*				
Current, *n* (%)			32 (45.7)	27 (38.6)
Former, *n* (%)			41 (58.6)	45 (64.3)
*Medication*				
CNS medication, *n* (%)			36 (51.4)	48 (68.6)
Sleep medication, *n* (%)			19 (27.1)	19 (27.1)
Other medication, *n* (%)			28 (40.0)	38 (54.3)
Comorbidities				
Physical illnesses, *n* (%)			46 (65.7)	54 (77.1)
*Psychological diagnoses*				
Current, *n* (%)			41 (58.6)	42 (60.0)
Thereof major depression, *n* (*%*)			37 (90.2)	41 (97.6)
Former, *n* (%)			35 (50.0)	42 (60.0)
Thereof major depression, *n* (*%*)			26 (74.3)	35 (83.3)
Outcomes at Baseline				
Depressive symptoms, *M* (*SD*)			16.37 (3.76)	16.80 (4.81)
Insomnia symptoms, *M* (*SD*)			19.94 (3.14)	19.21 (3.33)
Daytime sleepiness, *M* (*SD*)			8.46 (4.78)	8.79 (4.86)
Fatigue, *M* (*SD*)			5.65 (0.87)	5.58 (1.01)
* Well-being*, *M* (*SD*)			4.89 (2.54)	4.77 (2.80)
*Emotional state*				
Positive affect, *M* (*SD*)			20.19 (5.18)	19.64 (5.53)
Negative affect, *M* (*SD*)			29.31 (6.81)	27.79 (7.20)
*Emotion regulation*				
Self-focused rumination, *M* (*SD*)			18.39 (3.53)	18.09 (3.65)
Symptom-focused rumination, *M* (*SD*)			22.87 (4.41)	22.57 (4.19)
Distraction, *M* (*SD*)			17.21 (4.06)	16.97 (3.62)
Self-efficacy, *M* (*SD*)			2.95 (1.03)	2.98 (1.17)

*Note: M* and *SD* refer to means and standard deviations, respectively. *n* refers to the number of participants.

**Table 2:** Between-group effects of dCBT-I versus WLC group on primary and secondary outcomes.

Outcome	dCBT-I		WLC group		Diff_adj_	*p*	95% CI		*ES*
	*M*	*SD*	*M*	*SD*					
Depressive symptoms									
*Week 12*	11.22	5.75	14.87	5.15	−3.34	**<0.001**	−4.70	−1.98	−0.78
*Week 24*	12.10	5.64	14.98	5.25	−2.83	**<0.001**	−4.23	−1.43	−0.66
Insomnia symptoms									
*Week 12*	12.02	5.56	17.37	3.64	−6.26	**<0.001**	−7.57	−4.95	−1.94
*Week 24*	13.30	5.38	17.12	4.89	−4.72	**<0.001**	−6.06	−3.38	−1.46
Daytime sleepiness									
*Week 12*	8.81	4.96	9.39	4.96	0.28	0.607	−0.78	1.33	0.06
*Week 24*	8.90	5.22	9.22	4.50	0.20	0.721	−0.88	1.27	0.04
Fatigue									
*Week 12*	4.63	1.16	5.44	1.03	−0.92	**<0.001**	−1.21	−0.62	−0.98
*Week 24*	4.83	1.33	5.49	1.12	−0.76	**<0.001**	−1.05	−0.46	−0.81
Well-being									
*Week 12*	9.60	5.01	6.56	4.05	2.91	**<0.001**	1.72	4.10	1.09
*Week 24*	9.14	4.81	5.90	4.26	3.05	**<0.001**	1.83	4.26	1.14
Emotional state – Positive affect									
*Week 12*	25.70	6.79	22.54	6.44	2.99	**<0.001**	1.22	4.76	0.56
*Week 24*	25.60	7.17	20.45	6.11	5.26	**<0.001**	3.45	7.08	0.98
Emotional state – Negative affect									
*Week 12*	24.04	7.55	27.56	7.78	−3.67	**<0.001**	−5.59	−1.75	−0.52
*Week 24*	24.36	7.28	28.22	8.32	−5.17	**<0.001**	−7.15	−3.19	−0.74
Emotion regulation – Self-focused rumination									
*Week 12*	17.19	3.94	16.81	3.90	0.24	0.594	−0.65	1.14	0.07
*Week 24*	16.46	3.27	17.57	3.66	−1.17	**0.013**	−2.09	−0.25	−0.33
Emotion regulation – Symptom-focused rumination									
*Week 12*	20.32	4.65	21.87	5.04	−1.73	**0.005**	−2.95	−0.51	−0.40
*Week 24*	20.46	5.01	21.77	4.59	−1.97	**0.002**	−3.22	−0.72	−0.46
Emotion regulation – Distraction									
*Week 12*	17.64	3.51	17.62	3.66	−0.04	0.925	−0.96	0.87	−0.01
*Week 24*	18.02	3.71	17.60	3.72	0.23	0.627	−0.71	1.17	0.06
Self-efficacy									
*Week 12*	3.84	1.34	3.83	1.39	−0.02	0.932	−0.46	0.42	−0.02
*Week 24*	3.90	1.64	3.80	1.50	0.07	0.764	−0.37	0.51	0.06

*Note:* Diff_adj_, adjusted mean difference derived from linear mixed model; 95% CI, 95% confidence interval of the adjusted mean difference; *ES*, effect size (Cohen's *d*). Significant *p*-values are displayed in bold. *M* and *SD* refer to unadjusted means and standard deviations, respectively.

**Table 3:** Between-group effects of dCBT-I and WLC on sleep diary parameters.

Outcome	dCBT-I *n*=69*⁣*^*∗*^		WLC group *n*=70		Diff_adj_	*p*	95% CI		*ES*
	*M*	*SD*	*M*	*SD*					
SOL									
*Week 0*	48.16	29.76	52.18	35.90	—	—	—	—	—
*Week 12*	37.34	42.91	41.36	34.15	−6.00	0.207	−15.34	9.25	−0.18
*Week 24*	41.45	35.01	40.20	28.23	0.75	0.880	−9.06	3.34	0.02
WASO									
*Week 0*	40.86	35.98	31.98	33.63	—	—	—	—	—
*Week 12*	24.11	25.23	21.52	23.59	−10.17	**0.003**	−16.96	−3.38	−0.29
*Week 24*	23.66	21.36	26.90	33.28	−15.16	**<0.001**	−22.24	−8.09	−0.44
SE									
*Week 0*	73.38	10.46	77.12	10.67	—	—	—	—	—
*Week 12*	82.09	9.80	80.04	9.85	6.62	**<0.001**	3.94	9.29	0.63
*Week 24*	80.16	10.98	80.09	9.41	4.18	**0.004**	1.35	7.01	0.40
TST									
*Week 0*	390.09	64.24	406.87	72.27	—	—	—	—	—
*Week 12*	414.76	57.47	416.57	73.45	13.28	0.111	−3.06	29.61	0.19
*Week 24*	533.46	69.15	428.56	71.79	24.78	**0.005**	7.70	41.86	0.36
TIB									
*Week 0*	535.77	69.20	531.49	78.57	—	—	—	—	—
*Week 12*	509.19	64.85	523.28	77.67	−30.39	**0.002**	−49.72	−11.07	−0.41
*Week 24*	537.75	88.16	532.48	72.26	−3.32	0.746	−23.47	16.83	−0.04

*Note*: Diff_adj_, adjusted mean difference derived from linear mixed models; 95% CI, 95% confidence interval of the adjusted mean difference; ES, between-group effect size (Cohen's *d*). *M* and *SD* refer to unadjusted means and standard deviations, respectively. Week 0 refers to the baseline measurement. Significant *p*-values are displayed in bold.

Abbreviations: dCBT-I, digital cognitive behavioral therapy for insomnia; SE, sleep efficiency; SOL, sleep onset latency; TST, total sleep time; WASO, wake after sleep onset.

*⁣* ^*∗*^One participant had to be excluded due to a faulty diary.

## Data Availability

The data that support the findings of this study are available from the corresponding author upon reasonable request.
